# Comprehensive Characterization of the Function of Metabolic Genes and Establishment of a Prediction Model in Breast Cancer

**DOI:** 10.1155/2022/3846010

**Published:** 2022-04-19

**Authors:** Ruijing Yu, Mengle Peng, Shuai Zhao, Zhongquan Wang, Yajie Ma, Xinyu Zhang, Xuefeng Lv, Shukai Wang, Shaotan Ju, Rongling Zhao, Qing Zhou, Wenping Lian

**Affiliations:** ^1^Department of Clinical Laboratory, Henan Provincial Third People's Hospital, Zhengzhou, 450006 Henan, China; ^2^Department of Neurology, Henan Provincial Third People's Hospital, Zhengzhou, 450006 Henan, China; ^3^Department of Medical Affair, Henan Provincial Third People's Hospital, Zhengzhou, 450006 Henan, China; ^4^Department of Clinical Laboratory, The First Affiliated Hospital of Zhengzhou University, Zhengzhou 450052, China; ^5^Rehabilitation Medicine, The First Affiliated Hospital of Zhengzhou University, Zhengzhou 450052, China

## Abstract

**Background:**

Breast cancer (BC) is a highly heterogeneous disease with high morbidity and mortality. Its subtypes may have distinctly different biological behaviors, clinical outcomes, and therapeutic responses. The metabolic status of BC tissue is closely related to its progress. Therefore, we comprehensively characterized the function of metabolic genes in BC and identified new biomarkers to predict BC patients' prognoses.

**Methods:**

Metabolic genes were identified by intersecting genes obtained from two published pieces of literature. The function of metabolic genes in BC was determined by extracting differentially expressed genes (DEGs), performing functional enrichment analyses, analyzing the infiltrating proportion of immune cells, and conducting metabolic subgroup analyses. A risk score model was constructed to assess the prognoses of BC patients by performing the univariate Cox regression, LASSO algorithm, multivariate Cox regression, Kaplan-Meier survival analyses, and ROC curve analyses in the training set. The prognostic model was then validated on the testing dataset, external dataset, the whole TCGA-BC database, and our clinical specimens. Finally, a nomogram was constructed for clinical prognostic prediction based on the risk score model and other clinicopathological parameters.

**Results:**

955 metabolic genes were obtained. Among these, 157 metabolic DEGs were identified between BC and normal tissues for subsequent GO and KEGG pathway enrichment analyses. 5 metabolic genes were negatively correlated with CD8^+^ T cells, while 49 genes were positively correlated with CD8^+^ T cells. Furthermore, 5 metabolic subgroups with varying proportions of PAM50 subtypes, TNM classification, and immune cell infiltration were obtained. Finally, a risk score model was constructed to predict the prognoses of BC patients, and a nomogram incorporating the risk score model was established for clinical application.

**Conclusion:**

In this study, we elucidated tumor heterogeneity from metabolite profiling of BC. The roles of metabolic genes in the occurrence of BC were comprehensively characterized, clarifying the relationship between the tumor microenvironment (TME) and metabolic genes. Meanwhile, a concise prediction model was also constructed based on metabolic genes, providing a convenient and precise method for the individualized diagnosis and treatment of BC patients.

## 1. Introduction

The latest data shows that breast cancer (BC) was the most common type of cancer worldwide in 2020, with an estimated 2.3 million new cases. Although comprehensive efforts have been made in BC treatment, including surgery, chemotherapy, hormonal therapy, and radiation therapy, the outcome of BC patients is still poor. Moreover, BC is the fifth leading cause of cancer mortality worldwide, with 685,000 deaths [1, 2]. Meanwhile, BC is a highly heterogeneous disease whose subtypes may have distinctly different biological behaviors, clinical outcomes, and therapeutic responses [3]. Therefore, it is urgent to explore new prognostic factors to accurately judge the prognoses of BC patients.

Metabolism is an indispensable biochemical process in living organisms, providing energy and building blocks for macromolecules [4]. Through metabolic conversions, the cells can obtain metabolites and energy to survive. Those metabolic conversions are also involved in cell signaling and epigenetic networks [5, 6]. In addition, cancer cells reprogram their catabolic and anabolic metabolism to surpass nontumor cells, promoting proliferation, invasion, and metastasis [7]. In 1924, Dr. Otto Warburg observed cancer cells tended to perform glycolysis in the cytosol even in the presence of oxygen, which was known as the “Warburg effect” or “aerobic glycolysis” [8]. Since then, research has focused on the effects of tumor metabolism on tumor genesis and development, and metabolic reprogramming is now considered a hallmark of cancer [9]. Metabolic reprogramming directly and quickly supplies energy and nutrients for the proliferation of cancer cells in harsh survival conditions. It also shifts the tumor microenvironment (TME) into an immunosuppressive state by disrupting the metabolism and function of other TME components through multiple mechanisms, further inducing tumor progression [10, 11].

The metabolic status of BC tissue is also closely related to the progress of BC. Previous studies demonstrated that BC cells transitioning to the metastatic state displayed increased oxidative phosphorylation (OXPHOS) and glycolysis [12, 13]. Furthermore, BC cells induce oxidative stress and HIF-1*α* in adjacent fibroblasts, resulting in mitophagy and increased aerobic glycolysis [14]. Tumor cell metabolism produces several small molecule metabolites which can inhibit tumor immunity, such as lactic acid, adenosine, and kynurenine [11, 15]. For example, BC cell-derived lactate activates GPR81 in dendritic cells and prevents the presentation of tumor-specific antigens to other immune cells [16]. Cell-intrinsic factors causing metabolic reprogramming are considered oncogenes and tumor suppressor genes that regulate metabolic pathways at multiple levels [17]. In recent years, many metabolic genes have been reported to promote the development of BC, such as hexokinase (HK) and lactate dehydrogenase-A (LDHA), the two glycolysis-related genes highly expressed in BC [18, 19]. With the development of high-throughput sequencing, many databases have been established to research the genomic alterations of diseases. This enables the identification of biomarkers for prognostic prediction of patients, serving as potential clinical therapeutic targets [20–22].

In this study, we elucidated tumor heterogeneity through metabolite profiling of BC. The roles of metabolic genes in the occurrence of BC were comprehensively characterized, clarifying the relationship between the tumor microenvironment (TME) and metabolic genes. Furthermore, a concise prediction model was also constructed based on metabolic genes, providing a convenient and precise method for the individualized diagnosis and treatment of BC patients.

## 2. Methods and Materials

### 2.1. Dataset Collection and Preprocessing

The gene expression profiles (fragments per kilobase of exon per million (FPKM)) and corresponding clinical data of BC patients were obtained from The Cancer Genome Atlas (TCGA) database (https://portal.gdc.cancer.gov/repository). The gene expression data were converted into transcripts per kilobase million (TPM) normalized counts [23], and the normalized gene expression data were log2-transformed for further analyses. Subsequently, the genes with an average gene expression of less than 1 or those expressed in less than 50% of all samples were filtered out. The GSE20685 dataset was downloaded from the Gene Expression Omnibus (GEO) database (https://www.ncbi.nlm.nih.gov/geo/) and was used as the external dataset for validation. Furthermore, metabolic genes were retrieved from the published literature [24, 25]. The intersected genes from the two downloaded gene sets were used as metabolic genes for subsequent analyses.

### 2.2. Extraction of Differentially Expressed Genes

The “limma” R package was used to identify the differentially expressed genes (DEGs) [26]. Genes with adjusted *P* value < 0.05 and |log2 − fold change (log2 FC)| > 1 were enrolled in. A volcano map was drawn with the “ggplot2” package to display DEGs.

### 2.3. Functional Enrichment Analyses

To investigate the biological functions and characterize the metabolic genes, the R package “clusterProfiler” was used to perform the Gene Ontology (GO) and Kyoto Encyclopedia of Genes and Genomes (KEGG) functional enrichment analyses [27, 28]. A cutoff criterion of FDR < 0.05 was applied. The GO terms contained three main processes: biological process (BP), molecular function (MF), and cell component (CC). This study mainly focused on molecular function.

### 2.4. Immune Characterization

To further elucidate the relationship between metabolic genes and different immune cells, we analyzed the gene transcriptome data of BC patients to estimate the proportion of immune cells [29]. Through applying single sample Gene Set Enrichment Analysis (ssGSEA) algorithms [30], CIBERSORT algorithm [31], microenvironment cell population (MCP) [32] algorithms, and xCell algorithm [33], the metabolic genes affecting the proportion of infiltrating CD8^+^ T cells were identified. The above estimations were carried out by the R packages “GSVA,” “CIBERSORT,” “MCP-Counter,” and “xCell”, respectively. The Spearman correlation of the CD8^+^ T cell fraction among four algorithms was calculated and visualized by the “ggcor” package.

### 2.5. Metabolism-Related Consensus Clustering for BC

We applied the “ConsensusClusterPlus” package to identify BC molecular subtypes. This algorithm, one of the unsupervised class discovery algorithms, defined “consensus” clustering by applying a specific clustering means to the random data subsets [34, 35]. The “pheatmap” package was utilized to display the consensus clustering heatmap.

### 2.6. Prognostic Model Establishment

The impact of metabolic genes on the prognoses of BC patients was explored. First, the BC patients from the TCGA database were randomly divided into training cohorts and testing cohorts in a 1 : 1 ratio. Next, the univariate regression analysis (*P* < 0.05) was performed to identify candidate metabolic genes related to overall survival (OS) in the training cohort. The least absolute shrinkage and selection operator (LASSO) algorithm was used to further filter prognosis-specific metabolic genes [36]. Finally, genes identified in the LASSO analysis were enrolled in the multivariate Cox regression analysis to assess their impact on the OS. The prognostic model was established by the following formula: *β*1 × mRNA level of  gene1 + *β*2 × mRNA level of  gene2 + ⋯+*βn* × mRNA level of genen, where *β* corresponded to the correlation coefficient. The risk score (RS) of every patient was calculated based on the prognostic model. The univariate and multivariate Cox regression analyses were carried out by using “survival” R packages, and the “glmnet” package was used for the LASSO regression analysis [37]. Univariate analysis and multivariate analysis of forest maps were constructed by R package “forestplot.”

### 2.7. Quantitative Real-Time PCR (qRT-PCR)

50 BC patients were enrolled from Henan Provincial Third People's Hospital and signed written informed consent. Clinicopathological features of 50 BC patients are shown in Supplementary Table 1. Total RNA was extracted with TRIzol reagent (Invitrogen Corporation, Carlsbad, CA, #A33250) and then used for cDNA synthesis by the Prime Script RT reagent kit with genomic DNA eraser (TaKaRa, Tokyo, Japan, #RR037A). SYBR Green Master Mix (TaKaRa) was applied for qRT-PCR assay. The primer sequences for selected genes are displayed in Supplementary Table 2 and were synthesized by the Shanghai Sangon Company.

### 2.8. Statistical Analysis

Statistical analyses were performed with R (version 3.6.1). *P* < 0.05 was considered statistically significant. The Kaplan-Meier method was used to assess the differences in survival time, and the difference between survival curves was evaluated by a log-rank test. The specificity and sensitivity of the risk score model were measured by calculating the area under the curves (AUC) of the time-dependent receiver operating characteristic (ROC) curve. The different expression of genes between tumor and normal groups was judged by paired *T*-test, and the correlation of gene expression in clinical specimens was evaluated by Pearson's correlation coefficient using GraphPad Prism 7 software (La Jolla, USA). The survival curve was drawn with the Kaplan-Meier method, and the log-rank test was used to evaluate the difference between survival curves. The R package “rms” was used to construct a nomogram.

## 3. Results

### 3.1. Identification and Characterization of Metabolic DEGs between Tumor and Adjacent Normal Tissues

**Figure 1:**
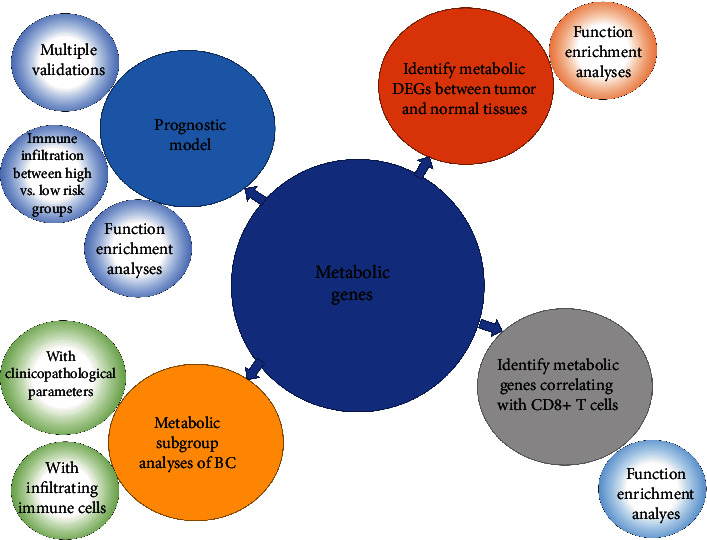
Graphical abstract of the study.

**Figure 2:**
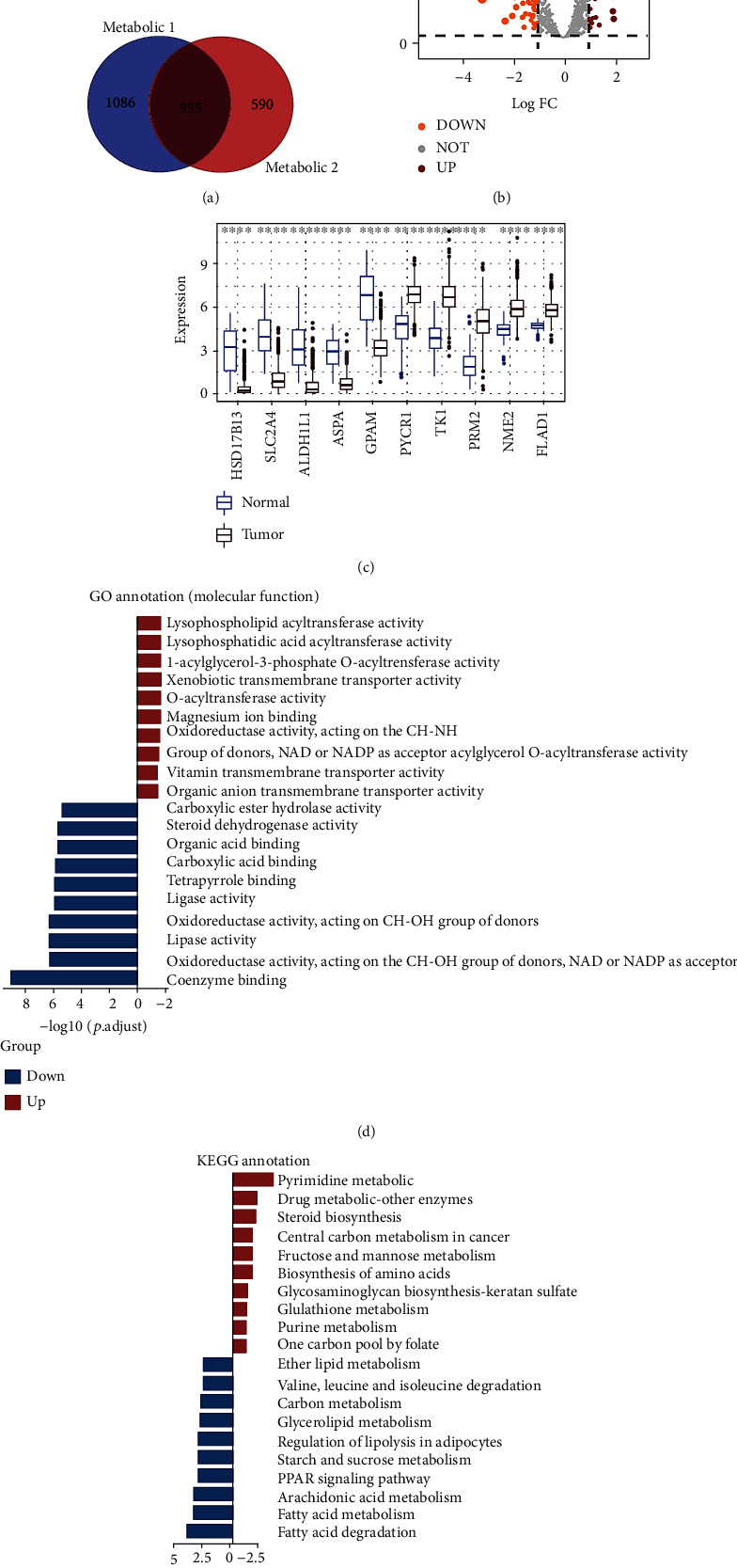
Identification of metabolic genes in BC patients between tumor and normal tissues. (a) Venn diagram showed the intersected metabolic genes. (b) Metabolic DEGs between tumor and normal tissues were demonstrated by volcano map. (c) Box chart displayed the top ten genes with the highest significance according to adjusted *P* value. (d) Top 10 most enriched molecular functions in GO analyses. (e) Top 10 most enriched KEGG pathway enrichment analyses.

As depicted by the graphical abstract, the metabolic heterogeneity of BC was comprehensively investigated from different aspects (Figure 1). Firstly, 955 intersected genes were obtained from two published pieces of literature for subsequent analyses (Figure 2(a)). The metabolic DEGs between BC and adjacent noncancerous tissues from the TCGA datasets were compared, revealing 46 upregulated genes and 111 downregulated genes in BC tissues (Figure 2(b)). Subsequently, the top 10 genes with the highest significance were displayed (Figure 2(c)), and we validated the top 3 genes (hydroxysteroid 17-beta dehydrogenase 13 (HSD17B13), solute carrier family 2 member 4 (SLC2A4), and aldehyde dehydrogenase 1 family member L1 (ALDH1L1)) based on adjusted *P* value in our clinical specimens, and the findings were consistent with the above results (Supplementary Figure 1A). GO (molecular function) analyses showed that upregulated genes were mainly focused on lysophospholipid acyltransferase activity and the downregulated genes were involved in coenzyme binding (Figure 2(d)). The results of KEGG pathway enrichment analyses indicated that upregulated genes were closely related to pyrimidine metabolism and downregulated genes were mainly enriched in fatty acid degradation (Figure 2(e)). These results indicate that the tumor tissues have different metabolic patterns as compared with normal tissues.

### 3.2. Identification and Characterization of Metabolic Genes Correlating with CD8^+^ T Cells

**Figure 3:**
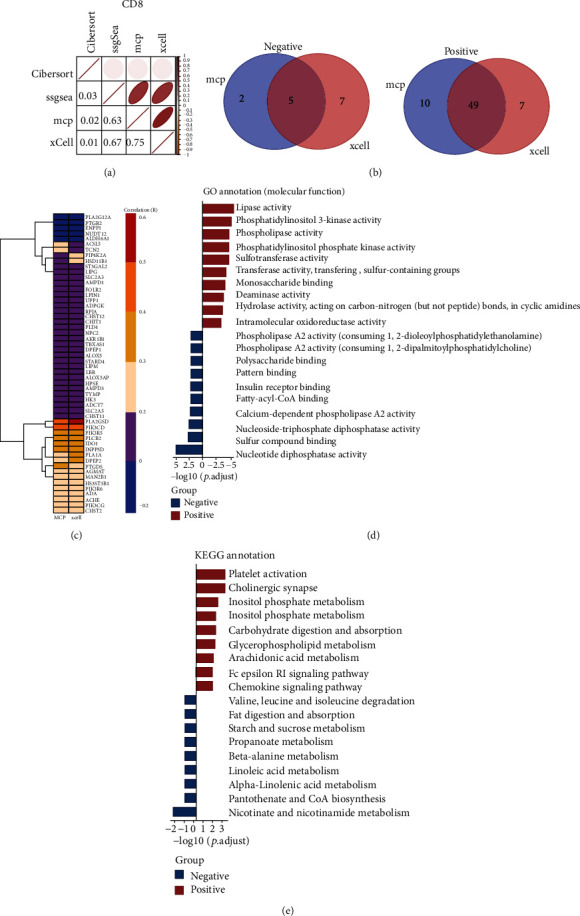
Identification and characterization of metabolic genes correlated with CD8^+^ T cells. (a) Correlation matrix of four algorithms based on the calculated scores of CD8^+^ T cells. (b) Venn diagrams showed the intersected genes. (c) Heatmap demonstrated relative correlation between 54 genes and CD8^+^ T cells through MCP algorithms and xCell algorithm. (d) Top 10 most enriched molecular functions in GO analyses. (e) Top 10 most enriched KEGG pathway enrichment analysis.

CD8^+^ T cells in the TME are manipulated by tumor cells through several mechanisms, including metabolic reprogramming. Therefore, four algorithms were applied to score CD8^+^ T cells for correlation analyses. There were high correlations among the ssGSEA algorithm, MCP algorithm, and xCell algorithm, especially between the MCPs and xCell algorithm (the correlation coefficient was 0.75), indicating a relatively strong consistency (Figure 3(a)). Next, the metabolic genes correlated with CD8^+^ T cells by the above two algorithms were extracted, and the intersected genes were identified (*P* < 0.05, |correlation coefficient| > 0.2). There were 5 metabolic genes negatively correlated with CD8^+^ T cells, while 49 genes were positively correlated with CD8^+^ T cells (Figure 3(b)). A heatmap was drawn to visually and independently demonstrate the correlation between 54 genes and CD8^+^ T cells, further confirming the reliability of the genes obtained from the above two algorithms (Figure 3(c)).  Table 1 also shows the top 5 genes phospholipase A2 group IID (PLA2G2D), phosphatidylinositol-4,5-bisphosphate 3-kinase catalytic subunit delta (PIK3CD), indoleamine 2,3-dioxygenase 1 (IDO1), inositol polyphosphate-5-phosphatase D (INPP5D), and phospholipase C beta 2 (PLCB2) which were correlated with CD8^+^ T cells based on xCell algorithm and MCP algorithm. We also verified the top 3 genes (PLA2G2D, PIK3CD, and IDO1) with the most significant *P* value in our clinical specimens and have found the consistent results (Supplementary Figure 1B). Moreover, GO analysis was performed to explore the molecular functions of those intersected genes. The results revealed that the genes positively correlated with CD8^+^ T cells were mainly involved in lipase activity, while the negatively correlated genes focused on nucleotide diphosphatase activity (Figure 3(d)). KEGG analyses indicated that the genes positively correlated with CD8^+^ T cells mainly focused on platelet activation, while the genes negatively correlated with CD8^+^ T cells were involved in nicotinate and nicotinamide metabolism (Figure 3(e)).

### 3.3. Metabolic Subgroup Analysis of BC

**Figure 4:**
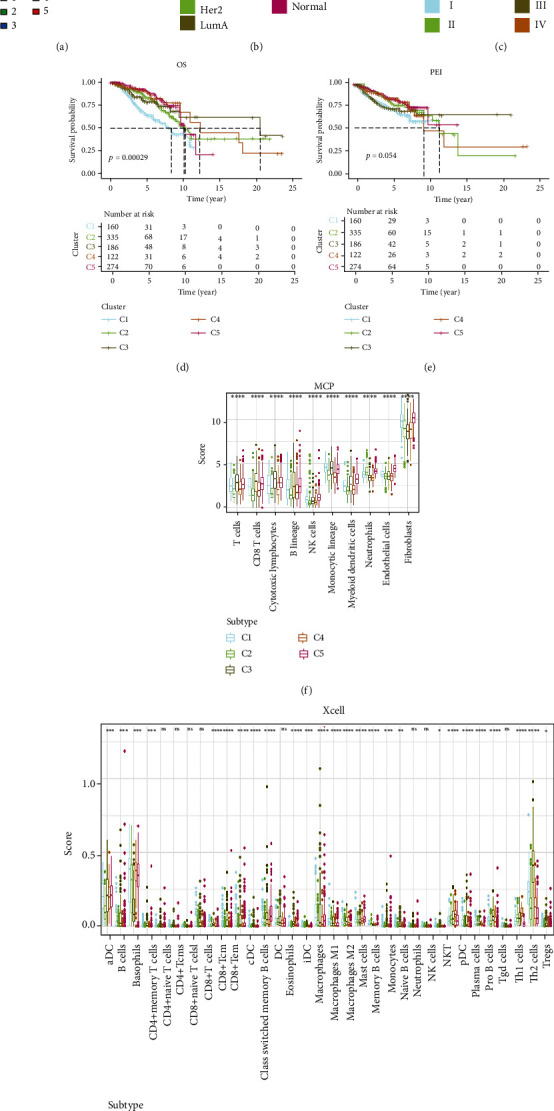
Consensus clustering of metabolic genes in TCGA-BC dataset. (a) Consensus matrices of metabolic genes for *k* = 5. (b) Bar charts of PAM50 subtype proportions among different metabolic subgroup patients. (c) Bar charts of TNM stage subtype proportions among different metabolic subgroup patients. (d) Kaplan-Meier curves for the OS of BC patients among different metabolic subgroups. (e) Kaplan-Meier curves for the PFI of BC patients among different metabolic subgroups. (f) The infiltrating immune cells of metabolic subpopulations calculated by MCP algorithms. (g) The infiltrating immune cells of metabolic subpopulations calculated by xCell algorithms.

In order to further analyze the role of metabolic genes in BC, we divided BC patients from the TCGA database into 5 subgroups by employing the 955 metabolic genes for consensus clustering analysis (Figure 4(a)). We next characterized the components of metabolic subgroups through PAM50 subtypes and found that LumA type had the highest proportion in the C1 subgroup, while C3 subgroup was mainly composed by basal type (Figure 4(b)). The metabolic subgroup findings were then compared with the tumor-node-metastasis (TNM) classification. We observed that the C3 subgroup had the smallest proportion of stage III and IV (Figure 4(c)), N2+N3 stage (Supplementary Figure 2A), a smaller proportion of T3+T4 stage (Supplementary Figure 2B), and M1 stage (Supplementary Figure 2C). Then, survival analyses showed the C3 subgroup had a better prognosis, both in terms of OS and progression-free interval (PFI) (Figures 4(d) and 4(e)). We further analyzed the immune cell infiltration of metabolic subpopulations and found higher infiltration in the C3 subpopulation, especially CD8^+^ T cells, but had fewer fibroblasts (Figures 4(f) and 4(g)), indicating that immune response may contribute to favorable survival of C3 subgroup.

### 3.4. Construction and Validation of Prognostic Model

**Figure 5:**
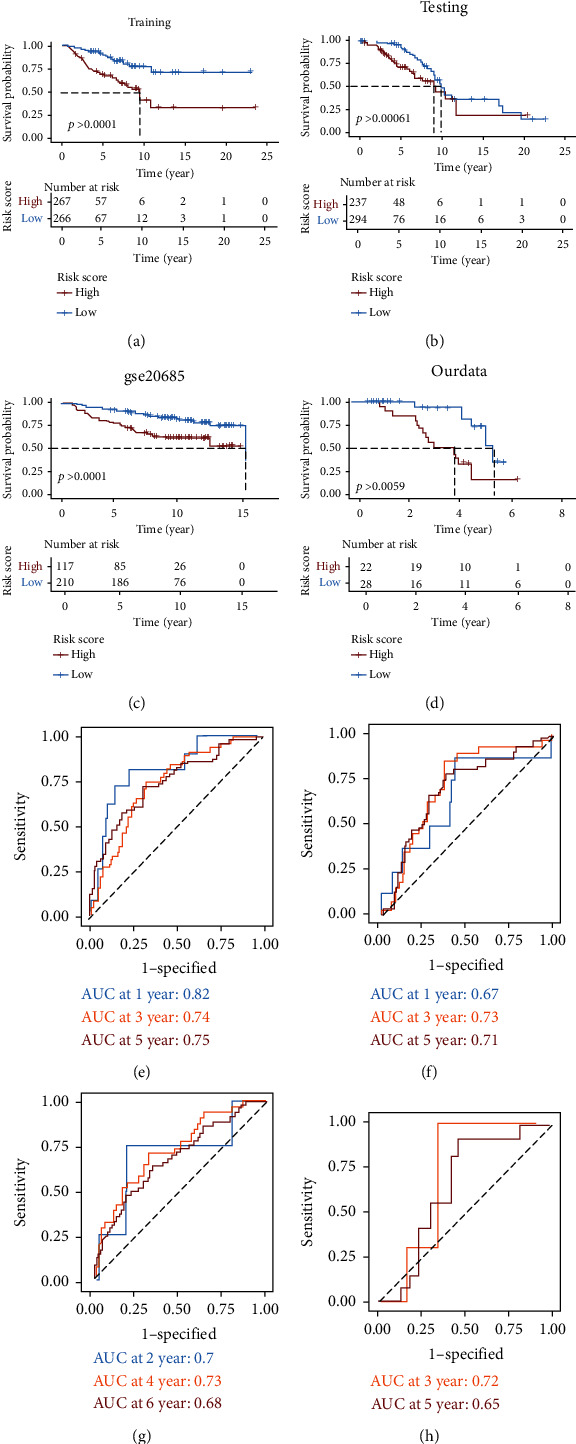
Five-gene metabolic signature is a prognostic biomarker for OS in different datasets. (a–d) KM survival analyses of OS for training, testing, external datasets, and the dataset based on our clinical specimens, respectively. (e–h) Time-dependent ROC curves of OS for training, testing, external datasets, and the dataset based on our clinical specimens, respectively.

To further explore the function of these metabolic genes in predicting survival of BC, the candidate genes were screened by the univariate Cox regression in the training set, and survival-related genes were further enrolled in the LASSO algorithm to establish an optimal prediction model. The 5 genes carboxyl ester lipase (CEL), phosphoglycerate kinase 1 (PGK1), iodotyrosine deiodinase (IYD), quinolinate phosphoribosyltransferase (QPRT), and solute carrier family 27 member 2 (SLC27A2) were chosen for the construction of the risk characteristic formula (Supplementary Figure 3A-B). They can all be used as independent prognostic predictors for BC patients (Supplementary Figure 3C-D). The final risk score model formula was constructed by a multivariate Cox regression: risk score = 0.196 × mRNA level of CEL + 0.530 × mRNA level of PGK1 + 0.279 × mRNA level of IYD + 0.187 × mRNA level of QPRT − 0.164 × mRNA level of SLC27A2. According to the predictive model, each BC patient was given a risk score, and the patients from different datasets were divided into high-/low-risk groups based on the optimal cutoff point. In the training dataset, patients in the high-risk group demonstrated a shorter survival time than patients in the low-risk group (Figure 5(a)). The above conclusion was then verified on the testing dataset (Figure 5(b)), external dataset (Figure 5(c)), the whole TCGA-BC dataset (Supplementary Figure 3E), and our clinical specimens (Figure 5(d)). The results were consistent with the above conclusion that patients with higher scores had a poorer prognosis. The predictive efficacy of our prognostic model was tested by ROC curve analyses, indicating our risk score model was accurate and sensitive. As illustrated by Figure 5(e), AUC results for 1-, 3-, and 5-year overall survival predictions in the training dataset were 0.82, 0.74, and 0.75, respectively. In the testing dataset, AUC results for 1-, 3-, and 5-year overall survival predictions were 0.67, 0.73, and 0.71, respectively (Figure 5(f)). In the external dataset, the AUCs for 2-, 4-, and 6-year overall survival predictions were 0.7, 0.73, and 0.68, respectively (Figure 5(g)). In the whole TCGA-BC database, AUC results for 1-, 3-, and 5-year overall survival predictions were 0.76, 0.73, and 0.73, respectively (Supplementary Figure 3F). Furthermore, the predictive efficiency was tested on our clinical specimens, and the AUCs for 3- and 5-year overall survival predictions were 0.72 and 0.65 (Figure 5(h)). Finally, the BC patients from the whole TCGA-BC dataset were divided into different subgroups according to age, pathological T stage, pathological N stage, pathological M stage, TNM stage, PAM50 classification, and risk score model and found that the model was effective in assessing the outcomes of BC patients (Supplementary Figure 4).

### 3.5. Clinical Application of a Nomogram Incorporating the Risk Score Model

**Figure 6:**
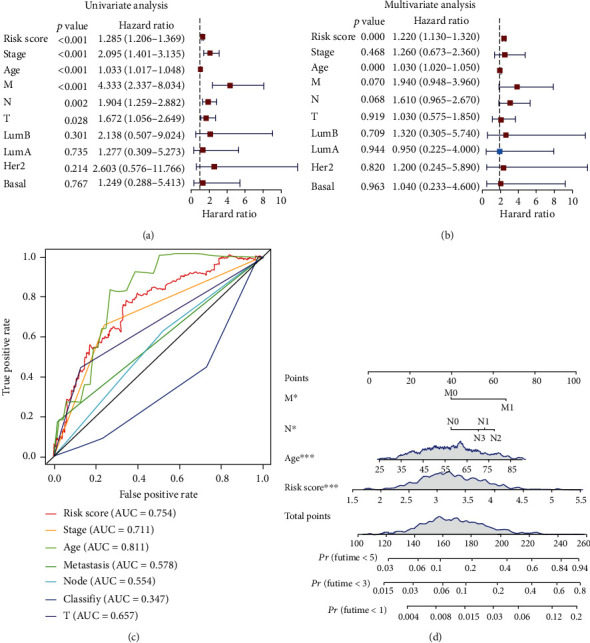
Construction of nomograms. (a) Forest plot shows the univariate Cox analysis of OS-related variables. (b) Forest plot shows the multivariate Cox analysis of OS-related variables. (c) The ROC curve analysis of OS-related variables. (d) Establish a nomogram to predict the OS of BC patients. The variables with *P* value < 0.1 were enrolled in.

In order to establish a more convenient and objective model that can be applied in clinical practice, a nomogram was constructed to evaluate the prognostic ability of our model in the BC patients from the TCGA database. Our risk model and several clinicopathological characteristics were enrolled into the univariate Cox regression analysis, revealing that the risk score (HR = 1.285, *P* < 0.001), TNM stage (HR = 2.095, *P* < 0.001), age (HR = 1.033, *P* < 0.001), pathological M stage (HR = 4.333, *P* < 0.001), pathological N stage (HR = 1.904, *P* = 0.002), and pathological T stage (HR = 1.672, *P* = 0.028) could predict the prognosis of BC patients (Figure 6(a)). The multivariable Cox regression analysis further confirmed that risk score (HR = 1.220, *P* < 0.0001) and age (HR = 1.030, *P* < 0.0001) could serve as independent prognostic biomarkers for BC patients (Figure 6(b)). The ROC curve analysis also indicated that the risk score model could accurately predict BC patients' outcomes (AUC = 0.754) (Figure 6(c)). Finally, we established a nomogram consisting of the above variables, which could quantitatively score the probability of BC patients' mortality. This tool provides a convenient method for the precise diagnosis and treatment of BC patients. Each patient is assigned a total score from the nomogram, where a higher risk score was associated with a poor prognosis (Figure 6(d)).

### 3.6. Immune and Genomic Alterations between High- and Low-Risk Groups

**Figure 7:**
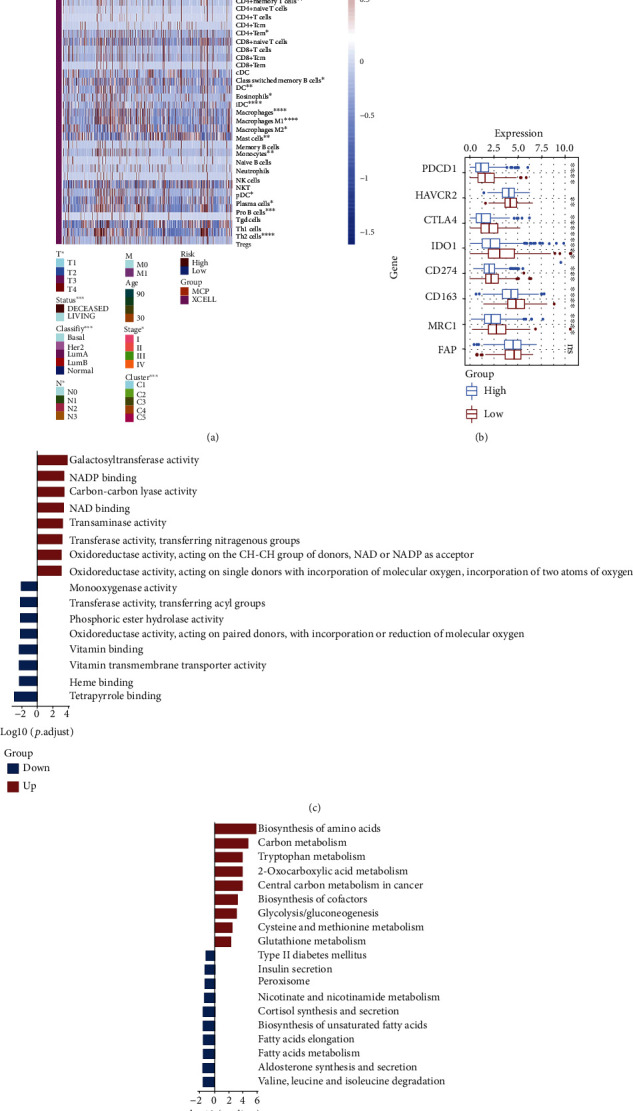
Investigation of immune infiltration between high- and low-risk groups. (a) Heatmap demonstrated the differences between high-/low-risk groups in immune infiltration and clinical parameters. (b) The expression of immunosuppressive molecules between high-/low-risk groups. (c) Top 10 most enriched molecular functions in GO analyses between high- and low-risk groups. (d) Top 10 most enriched KEGG pathway enrichment analyses between high- and low-risk groups.

The above demonstrated that the risk score model is effective in predicting the prognosis of BC patients and patients with high risk have worse survival. In order to further explore the causes behind the poorer prognoses, the differences in clinicopathological parameters between the high- and low-risk groups were analyzed. As shown in Figure 7(a), the TNM classification, PAM50 subtypes, and our previous metabolic clustering subtypes were significantly different in high- and low-risk groups. The MCP algorithm and xCell algorithm were employed to evaluate the immune cell infiltration in high- and low-risk groups. A higher degree of immune cell infiltration was found in the high-risk group, including T cells, monocytes, and neutrophils. Notably, immunosuppressive cells such as Th2 cells and M2 macrophages also exhibited a higher level of infiltration. Moreover, the high-risk group showed a higher expression of immunosuppressive molecules such as PDCD1, IDO1, CD274, and CD163, than the low-risk group (Figure 7(b)). The above implies that BC patients in the high-risk group may be exposed to an immunosuppressive TME, leading to tumor progression. By comparing patients in the high-risk group to the low-risk group, we extracted DEGs for functional enrichment analysis (Supplementary Figure 5). GO analysis revealed that upregulated DEGs were mainly involved in galactosyltransferase activity and downregulated DEGs were focused on tetrapyrrole binding (Figure 7(c)). KEGG analysis demonstrated that upregulated DEGs were mainly enriched in the biosynthesis of amino acids, while downregulated DEGs were centralized on the degradation of valine, leucine, and isoleucine (Figure 7(d)).

## 4. Discussion

BC is a disease with high morbidity and mortality and is also highly heterogeneous [1, 2, 38]. Patients with the same PMA50 classification or TNM stage may have different outcomes [39, 40]. Therefore, new biomarkers which have the potential to better predict BC patients' prognoses are urgently needed. Metabolic reprogramming has been considered as a hallmark of tumors, and metabolite profiling has become an informative approach to elucidate tumor heterogeneity [9, 24]. With the development of next-generation sequencing, many public transcriptomic databases have been established and have become convenient tools for oncology research [41]. Herein, we used public databases such as TCGA and GEO datasets to explore the metabolic reprogramming of BC.

Peng et al. demonstrated that metabolic expression subtypes indeed reflect metabolic activities and were associated with patients' survival [24]. Haider et al. reported that different metabolic requirements contributed to genomic heterogeneity and invasiveness among tumors [25]. In our study, we firstly extracted metabolic genes by intersecting gene sets downloaded from the above two pieces of literature. In order to further analyze the metabolic genes involved in BC tumorigenesis, metabolic DEGs between BC and adjacent noncancerous tissues were extracted from the TCGA datasets. Some metabolic DEGs had been reported to participate in the occurrence and development of breast tumors. Putluri N et al. found ribonucleotide reductase M2 (RRM2) as a prognostic marker in BC associated with poor survival and tamoxifen resistance [42]. One study had indicated that serum Thymidine kinase 1 (TK1) was higher in BC patients when compared with blood donors [43]. Ding et al. revealed that pyrroline-5-carboxylate reductase 1 (PYCR1) was associated with poor differentiation and aggressive phenotypes of BC [44]. The above previous studies were consistent with our study results. Functional enrichment analyses elucidated the function of metabolic genes in BC. GO analyses demonstrated that metabolic genes overexpressed in BC tumor tissues mainly focused on lysophospholipid acyltransferase activity, lending support to previous research. Lebok et al. revealed lysophosphatidylcholine acyltransferase 1 (LPCAT1) as one of the core genes in lysophospholipid acyltransferase activity was linked to poor prognosis in BC [45]. KEGG pathway enrichment indicated that the genes overexpressed in BC tumor tissues are mainly involved in pyrimidine metabolism. In contrast, the genes that were overexpressed in adjacent noncancerous tissues were mainly involved in the coenzyme binding by GO analyses and in fatty acid degradation by KEGG pathway analyses.

CD8^+^ T cells are a vital component of the adaptive immune system and play an essential role in host immune surveillance and immune clearance against tumors. Previous research has confirmed that the infiltration of CD8^+^ T cells in BC tissues was correlated with a better prognosis [46, 47]. CD8^+^ T cells in the TME are manipulated by tumor cells through several mechanisms, resulting in immune exhaustion and immune dysfunction, thus weakening the monitoring and clearance of tumor cells [48, 49]. Therefore, it is important to evaluate the function and infiltration of CD8^+^ T cells in BC tissues. We utilized the MCP algorithm and xCell algorithm to obtain metabolic genes correlated with CD8^+^ T cells and found 49 positively correlated genes and 5 negatively correlated genes with CD8^+^ T cells. The above genes can be employed to estimate the infiltration of CD8^+^ T cells. Surprisingly, some of the upregulated genes had been reported to be associated with T cell dysfunction. PIK3CD encodes the p110d isoform of the catalytic subunit of phosphoinositide 3-kinase (PI3K). Edwards et al. had reported germline gain-of-function (GOF) mutations in PIK3CD aberrantly induced exhaustion and impaired cytotoxicity of CD8^+^ T cells [50]. Several studies have reported that IDO1 could suppress the function of local CD8^+^ T effector cells [51]. We deduce that BC patients with high levels of these genes may suffer a “Hot” tumor status with high infiltration of exhausted CD8^+^ T cells [52, 53]. Biological function analyses revealed that the genes positively correlated with CD8^+^ T cells were closely related to metabolic processes, especially in lipase activity and platelet activation. In contrast, genes that were negatively correlated with CD8^+^ T cells were focused on nucleotide diphosphatase activity. This section may provide potential principles for screening the immunotherapy-adapted population.

We conducted consensus clustering analysis by employing metabolic genes and obtained 5 metabolic subgroups. PAM50 classification is a method characterized by 5 intrinsic molecular subtypes according to the microarray-based gene expression profiling of BC patients, including the luminal A (LumA), luminal B (LumB), HER2-enriched (HER2-E), basal-like, and normal-like breast cancer subtypes [39, 54]. Significant heterogeneity was observed within the components of PAM50 subtypes, especially between C1 and C3 subgroups. Based on the TNM classification, we also deduced that the other 4 subgroups were more aggressive than the C3 subgroup [40], and further survival analyses also revealed C3 subgroup had a better prognosis. Proportion analyses of infiltrating immune cells were performed to investigate the underlying mechanism. Higher infiltration with CD8^+^ T cells and less fibroblast infiltration were associated with better outcomes. These findings have also been reported by previous studies. BC patients with more CD8^+^ T cell infiltration [46, 49, 52] or less fibroblast infiltration [55] had better survival outcomes.

There are many well-established prediction models based on public transcriptomic databases. For example, Liu Y et al. identified a 22-autophagy gene signature based on TCGA and GEO lung cancer cohorts. They also confirmed that gene signature was an independent predictor of prognosis [56]. Through analyzing hepatocellular carcinoma (HCC) TCGA dataset, Zhang et al. showed that hypoxia-related signature is a potential biomarker for diagnosis, prognosis, and recurrence of HCC [57]. In our study, the univariate Cox regression, LASSO Cox regression, and multivariate Cox regression were further performed in training cohort, and a 5-gene signature was constructed for precise prognostic prediction of BC patients. Several previous studies have reported that the extracted genes individually affect the prognosis of BC patients. For example, Cui et al. described that high enzyme CEL expression might be an independent prognostic factor for poor survival of BC patients [58]. PGK1 had been reported as a major enzyme in the aerobic glycolysis pathway to induce cancer progression [59]. Liu et al. confirmed QPRT to enhance the invasiveness of BC probably through purinergic signaling and might be a potential prognostic indicator and therapeutic target in BC [60]. The above also demonstrated the prognostic value of our risk score model. Its accuracy was verified on different datasets of BC patients by survival analyses and ROC curve analyses. The results indicated that the risk score model could serve as a superior tool for predicting the prognosis of BC patients. Higher risk scores are associated with a poorer prognosis. We also obtained that in the subgroups classified by different pathological parameters, our risk score model could better judge patients' prognoses. The risk model can accurately predict BC patients' outcomes based on the clinicopathological parameters. Additionally, the univariate and multivariate Cox regression analyses proved that our risk score model can independently predict BC patients' survival. Furthermore, the nomogram integrating independent prognostic factors indicated that the risk score signature can stably and precisely predict BC patients' survival. This provides a clinically convenient prognostic assessment strategy. Finally, we observed that BC patients in the high-risk group exhibit an immunosuppressive TME due to the higher infiltration of immunosuppressive cells such as Th2 cells [61] and M2 macrophages [62], leading to poor prognoses for BC patients. Moreover, higher expression of immune checkpoints, such as PDCD1, IDO1, CD274, and CD163, was detected in the high-risk group.

## 5. Conclusions

In summary, this study comprehensively investigated the effects of metabolite profiles in BC patients. A 5-gene metabolic prognostic signature was constructed for precise diagnostic assessment and individualized treatment of BC patients.

## Figures and Tables

**Table 1:** Detailed information of top 5 genes which were correlated with CD8^+^ T cells.

Gene	xCell		MCP	
	Correlation (*R*)	*P* value	Correlation (*R*)	*P* value
PLA2G2D	0.621	<0.001	0.584	<0.001
PIK3CD	0.523	<0.001	0.522	<0.001
IDO1	0.493	<0.001	0.482	<0.001
INPP5D	0.467	<0.001	0.468	<0.001
PLCB2	0.453	<0.001	0.450	<0.001

## Data Availability

The data support the findings of this study are available from the corresponding author upon reasonable request.

## Supplementary Materials

Supplementary Materials Supplementary Figure 1: validations on our clinical specimens. (A) Validation of DEG expression between tumor and normal tissues. (B) Validation of identified metabolic genes that correlated with CD8^+^ T cells. Supplementary Figure 2: bar charts of TNM classification subtype proportions among different metabolic subgroup patients. (A) Pathological N stage. (B) Pathological T stage. (C) Pathological M stage. Supplementary Figure 3: establishment of prognostic model. (A-B) Identification of 5 metabolic genes by LASSO regression analysis. (C) 5 metabolic genes can individually predict the prognoses of BC patients. (D) A forest plot of hazard ratios for 5 metabolic genes. (E) Kaplan-Meier curves for the OS of patients deriving from the whole TCGA-BC cohort in low and high-risk groups. (F) ROC curves were used to assess the efficiency of the risk model for predicting 1-, 3-, and 5-year survival in the whole TCGA-BC cohort. Supplementary Figure 4: KM survival subgroup analyses of BRCA patients deriving from TCGA-BC database according to risk score model. (A) Age > 60. (B) Age ≤ 60. (C) Pathological T1+2 stage. (D) Pathological T3+4 stage. (E) Pathological N0 stage. (F) Pathological N1+2+3 stage. (G) Pathological M0 stage. (H) Early stage (stage I+II). (I) Late stage (stage III+IV). (J) Basal-like type in PAM50 classification. (K) HER-2-enriched type in PAM50 classification. (L) LumA type in PAM50 classification. (M) LumB-enriched type in PAM50 classification. (N) Normal breast-like type in PAM50 classification. Supplementary Figure 5: identification of metabolic genes in BC patients between high- and low-risk groups. (A) Metabolic DEGs between high- and low-risk groups were demonstrated by volcano map. (B) Heatmap demonstrated that metabolic DEGs between high- and low-risk groups. The upregulated DEGs were shown in red, while downregulated DEGs were shown in blue. Supplement Table 1: clinicopathological features of 50 BC patients from Henan Provincial Third People's Hospital. Supplementary Table 2: primers of genes used in this study.
