# Supplementary material for: Comprehensive Characterization of the Function of Metabolic Genes and Establishment of a Prediction Model in Breast Cancer
Source: Dis Markers. 2022 Apr 19;2022:3846010. doi: 10.1155/2022/3846010 (PMC9042645; doi:10.1155/2022/3846010)
Supplement: Supplementary Materials — Supplementary Figure 1: validations on our clinical specimens. (A) Validation of DEG expression between tumor and normal tissues. (B) Validation of identified metabolic genes that correlated with CD8+ T cells. Supplementary Figure 2: bar charts of TNM classification subtype proportions among different metabolic subgroup patients. (A) Pathological N stage. (B) Pathological T stage. (C) Pathological M stage. Supplementary Figure 3: establishment of prognostic model. (A-B) Identification of 5 metabolic genes by LASSO regression analysis. (C) 5 metabolic genes can individually predict the prognoses of BC patients. (D) A forest plot of hazard ratios for 5 metabolic genes. (E) Kaplan-Meier curves for the OS of patients deriving from the whole TCGA-BC cohort in low and high-risk groups. (F) ROC curves were used to assess the efficiency of the risk model for predicting 1-, 3-, and 5-year survival in the whole TCGA-BC cohort. Supplementary Figure 4: KM survival subgroup analyses of BRCA patients deriving from TCGA-BC database according to risk score model. (A) Age > 60. (B) Age ≤ 60. (C) Pathological T1+2 stage. (D) Pathological T3+4 stage. (E) Pathological N0 stage. (F) Pathological N1+2+3 stage. (G) Pathological M0 stage. (H) Early stage (stage I+II). (I) Late stage (stage III+IV). (J) Basal-like type in PAM50 classification. (K) HER-2-enriched type in PAM50 classification. (L) LumA type in PAM50 classification. (M) LumB-enriched type in PAM50 classification. (N) Normal breast-like type in PAM50 classification. Supplementary Figure 5: identification of metabolic genes in BC patients between high- and low-risk groups. (A) Metabolic DEGs between high- and low-risk groups were demonstrated by volcano map. (B) Heatmap demonstrated that metabolic DEGs between high- and low-risk groups. The upregulated DEGs were shown in red, while downregulated DEGs were shown in blue. Supplement Table 1: clinicopathological features of 50 BC patients from Henan Provincial Third People's Hospit [file 3846010.f1.docx]

# Supplementary Figure

**Supplementary Figure 1**


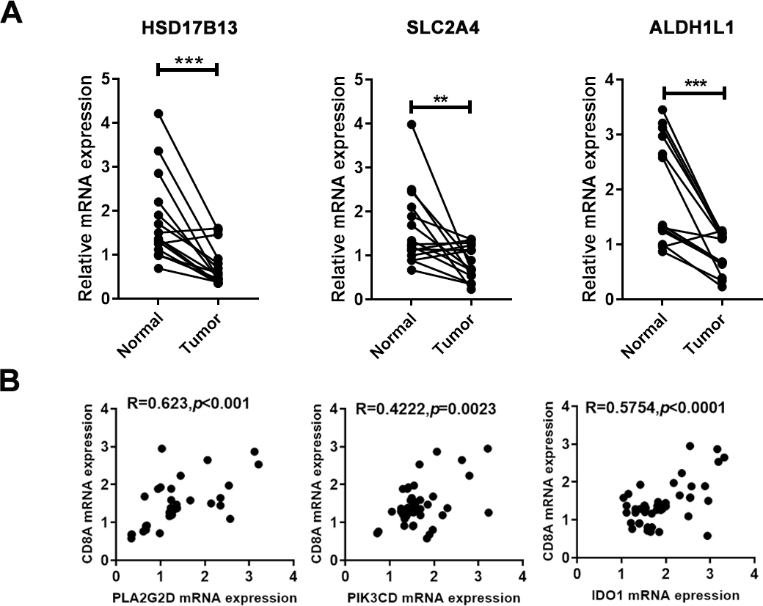


**Supplementary Figure 2**


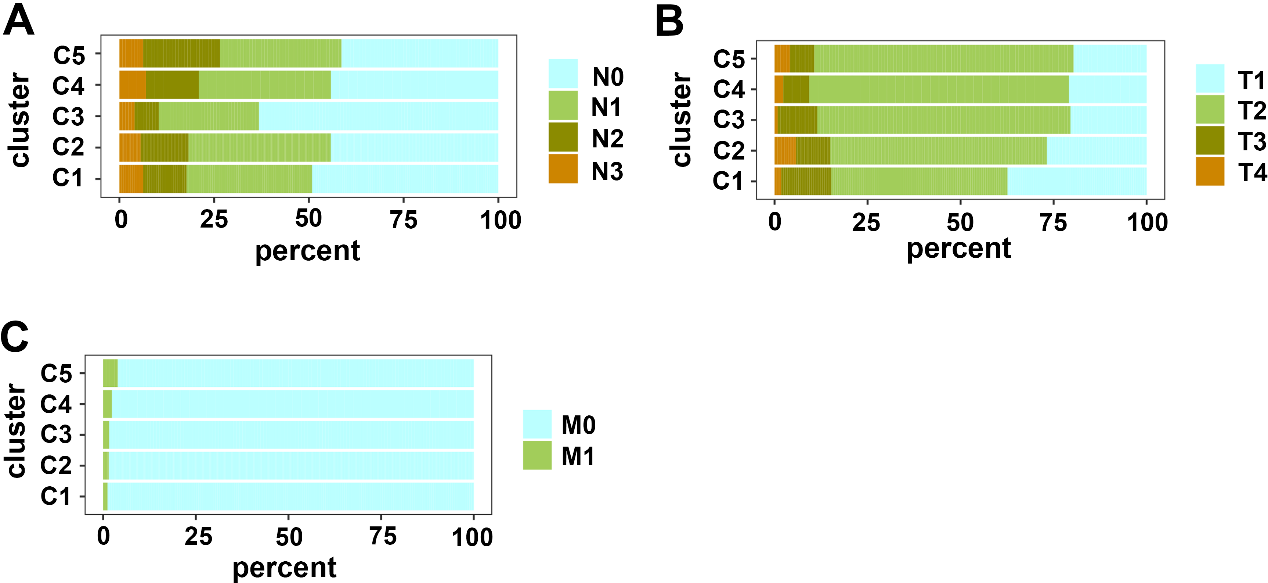


**Supplementary Figure 3**

**
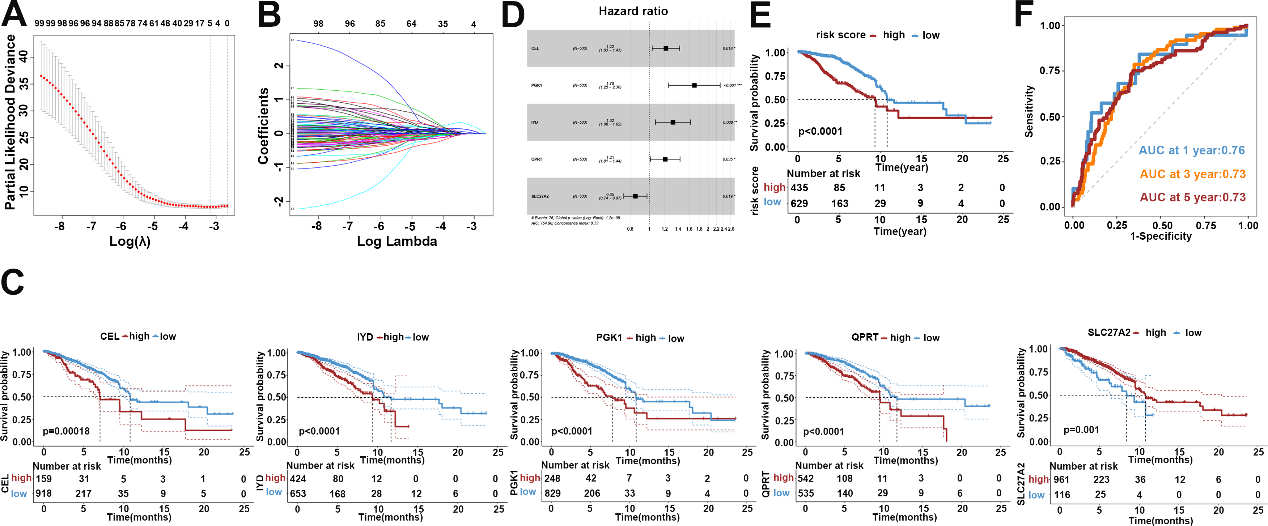
**

**Supplementary Figure 4**


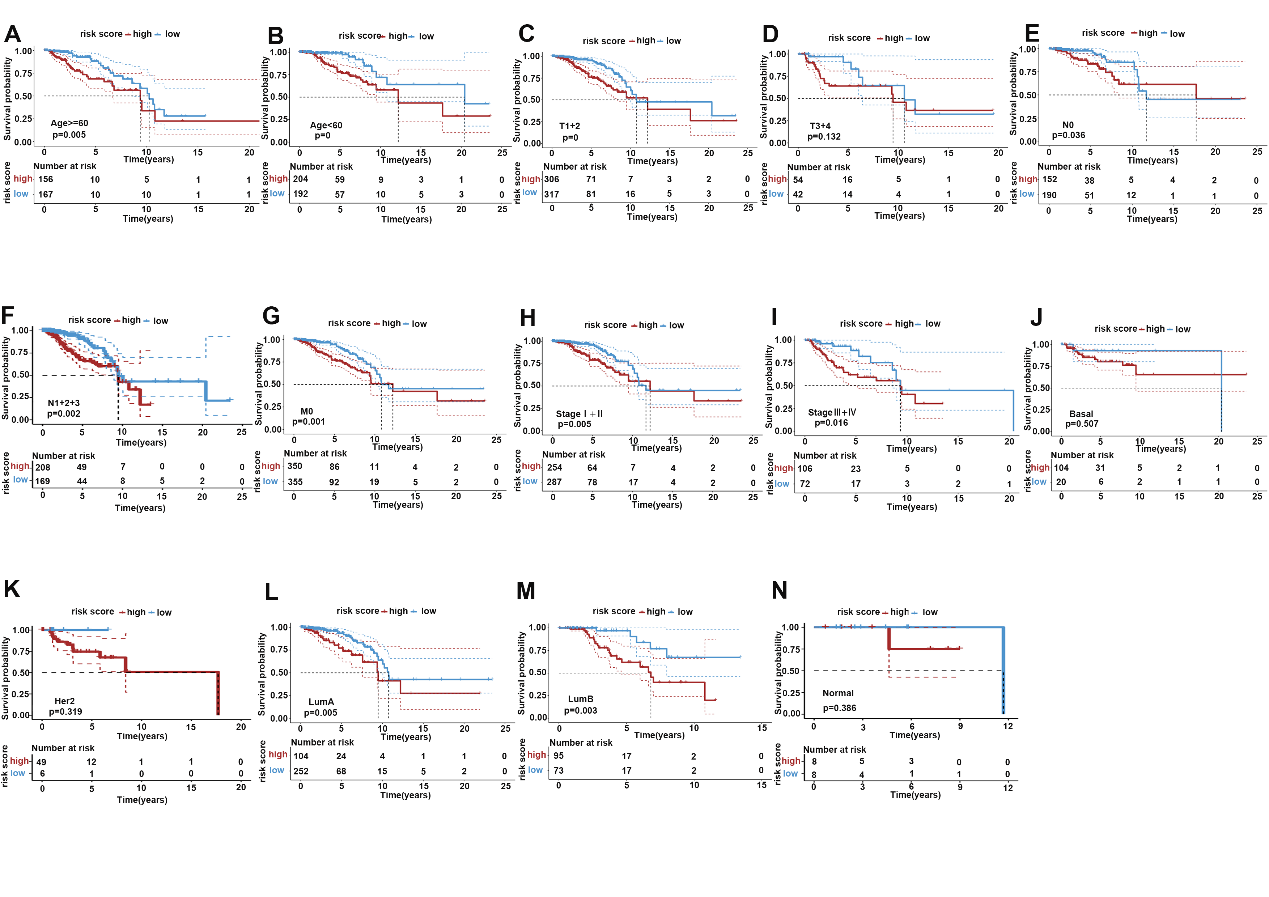


**Supplementary Figure 5**


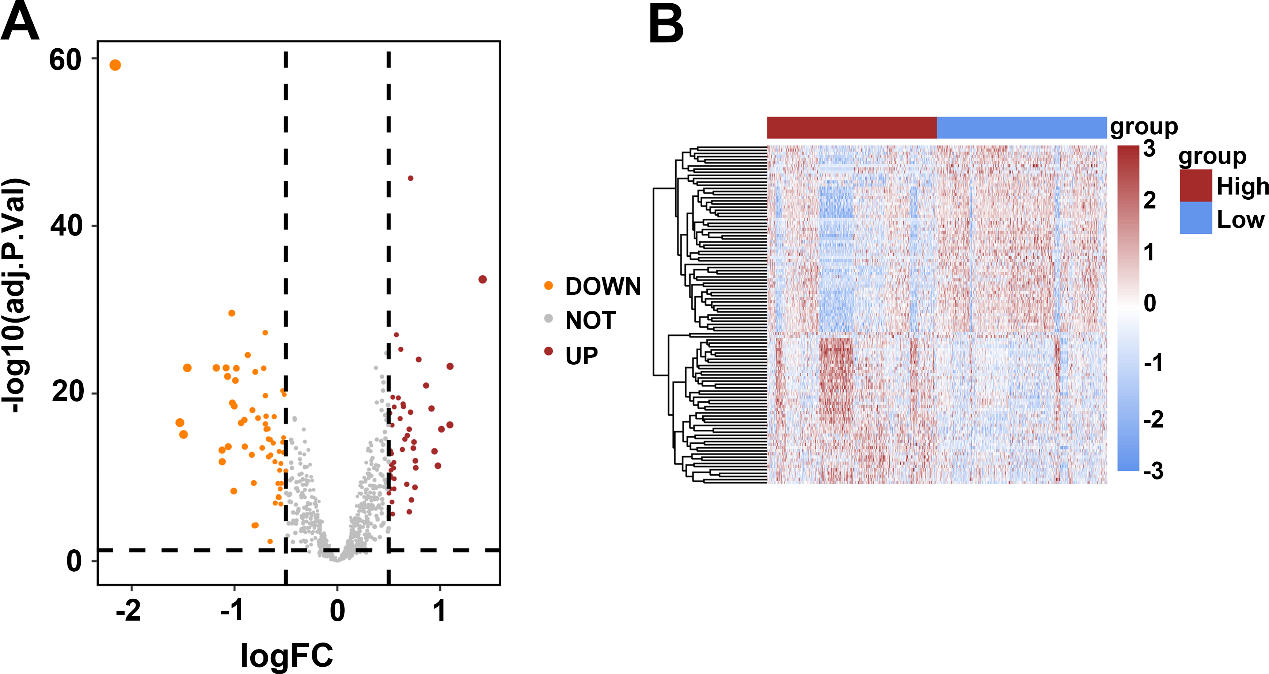


# Legends

**Supplementary Figure 1** Validations on our clinical specimens. **(A)** Validation of DEGs expression between tumor and normal tissues. **(B)** Validation of identified metabolic genes that correlated with CD8+T cells.

**Supplementary Figure 2** Bar charts of TNM classification subtypes proportions among different metabolic subgroup patients. **(A)** pathological N stage. **(B)** pathological T stage. **(C)** pathological M stage.

**Supplementary Figure 3** Establishment of prognostic model. **(A-B)** Identification of 5 metabolic genes by LASSO regression analysis. **(C)** 5 metabolic genes can individually predict the prognoses of BC patients. **(D)** A forest plot of hazard ratios for 5 metabolic genes. **(E)** Kaplan-Meier curves for the OS of patients deriving from the whole TCGA-BC cohort in low and high-risk groups. **(F)** ROC curves were used to assess the efficiency of the risk model for predicting 1‑, 3‑ and 5‑year survival in the whole TCGA-BC cohort.

**Supplementary Figure 4** KM survival subgroup analyses of BRCA patients deriving from TCGA-BC database according to risk score model. **(A)** Age >60. **(B)** Age ≤60. **(C)** Pathological T1+2 stage. **(D)** Pathological T3+4 stage. **(E)** Pathological N0 stage. **(F)** Pathological N1+2+3 stage. **(G)** Pathological M0 stage. **(H)** Early stage (stage I+II). **(I)** Late stage (stage III+IV). **(J)** Basal-like type in PAM50 classification. **(K)** HER-2 enriched type in PAM50 classification. **(L)** LumA type in PAM50 classification. **(M)** LumB enriched type in PAM50 classification. **(N)** Normal breast-like type in PAM50 classification.

**Supplementary Figure 5** Identification of metabolic genes in BC patients between high and low risk groups. **(A)** Metabolic DEGs between high and low-risk groups were demonstrated by volcano map. **(B)** Heatmap demonstrated that metabolic DEGs between high and low risk groups. The up-regulated DEGs were showed in red while down-regulated DEGs were showed in blue.

**Supplement Table 1: Clinicopathological features of 50 BC patients from** **Henan Provincial Third People’s Hospital**

| Characteristic | n |
| --- | --- |
| Age（years） |  |
| ≥60 | 28 |
| <60 | 22 |
| Gender |  |
| Male | 0 |
| Female | 50 |
| T stage |  |
| T1 | 5 |
| T2 | 30 |
| T3 | 13 |
| T4 | 2 |
| N stage |  |
| N0 | 14 |
| N1 | 15 |
| N2 | 14 |
| N3 | 7 |
| M stage |  |
| M0 | 45 |
| M1 | 5 |
| TNM stage |  |
| Stage 1 | 4 |
| Stage 2 | 18 |
| Stage 3 | 23 |
| Stage 4 | 5 |
|  |  |

**Supplementary Table2: Primers of genes used in this study**

| Gene | Forward | Reverse |
| --- | --- | --- |
| GAPDH | 5-GGAGCGAGATCCCTCCAAAAT-3 | 5-GGCTGTTGTCATACTTCTCATGG-3 |
| CEL | 5-GTCACCTTCAACTACCGTGTC-3 | 5-GGCCGCGATATTCCTCTTCAC-3 |
| PGK1 | 5-GACCTAATGTCCAAAGCTGAGAA-3 | 5-CAGCAGGTATGCCAGAAGCC-3 |
| IYD | 5-CCAGACGTGAAGCACAAGATT-3 | 5-GGATGCCACAAGCGATGGAA-3 |
| QPRT | 5-GGGCAGCCTTTCTTCGATG-3 | 5-GGAGCCCATACTTCTCCACCA-3 |
| SLC27A2 | 5-TTTCCGCCATCTACACAGTCC-3 | 5-CGTAGGTGAGAGTCTCGTCG-3 |
| HSD17B13 | 5-CCTACTTGGAGTCGTTGGTGA-3 | 5-CCAATATGCTCTGTCGTTTTGC-3 |
| SLC2A4 | 5-TGGGCGGCATGATTTCCTC-3 | 5-GCCAGGACATTGTTGACCAG-3 |
| ALDH1L1 | 5-GCTCCATCATCTATCACCCGT-3 | 5-ATCTCCGTGAATGAGGGTCCA-3 |
| CD8A | 5-ATGGCCTTACCAGTGACCG-3 | 5-AGGTTCCAGGTCCGATCCAG-3 |
| PLA2G2D | 5-AAAGATGCCACGGACTGGTG-3 | 5-CTTCTGGTAGGTGTCCAGGT-3 |
| PIK3CD | 5-TCAACTCACAGATCAGCCTCC-3 | 5-CGCGAAAGTCGTTCACTTCT-3 |
| IDO1 | 5-TGGGGCAAAGGTCATGGAG-3 | 5-TTTCTTGGAGAGTTGGCAGTAAG-3 |
